# Intervertebral disc and vertebral endplate subchondral changes associated with Modic 1 changes of the lumbar spine: a cross-sectional study

**DOI:** 10.1186/s12891-017-1407-6

**Published:** 2017-01-23

**Authors:** Christelle Nguyen, Marylène Jousse, Serge Poiraudeau, Antoine Feydy, François Rannou

**Affiliations:** 1 Université Paris Descartes, Sorbonne Paris Cité, 75006 Paris, France; 2 Service de Rééducation et de Réadaptation de l’Appareil Locomoteur et des Pathologies du Rachis, Hôpitaux Universitaires Paris Centre - Groupe Hospitalier Cochin, Assistance Publique - Hôpitaux de Paris, 75014 Paris, France; 3 INSERM UMR 1124, Laboratoire de Pharmacologie, Toxicologie et Signalisation Cellulaire, Faculté des Sciences Fondamentales et Biomédicales, 75006 Paris, France; 4 INSERM UMR 1153, 75004 Paris, France; 5 Institut Fédératif de Recherche sur le Handicap, 75013 Paris, France; 6 Service de Radiologie B, Hôpitaux Universitaires Paris Centre - Groupe Hospitalier Cochin, Assistance Publique - Hôpitaux de Paris, 75014 Paris, France; 7 Service de Rééducation et Réadaptation de l’Appareil locomoteur et des Pathologies du Rachis, Hôpitaux Universitaires Paris Centre-Groupe Hospitalier Cochin, 27, Rue du Faubourg Saint-Jacques, 75014 Paris, France

**Keywords:** Modic 1, MRI, Degenerative disc disease, Vertebral endplate subchondral bone, Active discopathy

## Abstract

**Background:**

Modic 1 changes are usually associated with degenerative disc disease (DDD). We aimed to compare Modic 1 changes with advanced degenerative disc disease (>50%-intervertebral space narrowing [IVSN]) to Modic 1 changes with less advanced lumbar degenerative disc disease (≤50%-IVSN).

**Methods:**

We conducted a cross-sectional study. The computerized MRI database from a French tertiary care hospital was searched. Patients were included if they were ≥ 18 years old and had a lumbar MRI between January 1, 2006 and January 31, 2008, that showed a Modic 1 signal at a single level. The strength of the magnet was 1.5 T. MRI were reviewed by 2 assessors. Age and gender were recorded. MRI changes involving the intervertebral disc and the vertebral endplate subchondral bone were assessed for Modic 1 signal, intervertebral space narrowing, asymmetrical degenerative disc disease, spondylolisthesis, anterior and posterior intervertebral disc herniation, and anterior and lateral osteophytes. These outcomes were compared between >50%-IVSN Modic 1 and ≤50%-IVSN Modic 1 groups. For bivariate analysis, comparisons involved nonparametric Kruskal-Wallis test for quantitative variables and nonparametric Fisher’s exact test for qualitative variables. Multivariate analysis was conducted to determine factors independently associated with <50%-IVSN Modic 1 changes by backward stepwise regression. Informed consent and formal approval from Institutional Review Board is not required for this type of study. This statement was confirmed by our Institutional Review Board.

**Results:**

MRI for 101 individuals were eligible. Patients’ mean (SD) age was 56.6(13.4) years, and 41/101(40.6%) were men. Modic 1 were most frequently observed at L4/L5 and L5/S1 (37[36.6%] cases each). As compared with >50%-IVSN Modic 1 patients, ≤50%-IVSN Modic 1 patients were younger (mean[SD] age 51.5[14.1] *vs* 58.8[12.6] years, *p* = 0.019), Modic 1 were more frequent at L5/S1 level (19[61.3%] *vs* 18[25.7%], *p* = 0.001), and anterior and lateral osteophytes were less frequent (13[41.9%] *vs* 55[78.6%], *p* < 0.001, and 11[35.5%] *vs* 48[68.6%], *p* = 0.002, respectively).

**Conclusions:**

≤50%-IVSN Modic 1 are rather found in young men at L5/S1 level and are associated with less frequent osteophytes than >50%-IVSN Modic, while >50%-IVSN Modic 1 are rather found in older women at L4/L5 level.

## Background

Modic 1 signal changes detected on magnetic resonance imaging (MRI) encompass clinical [1–3], radiological [4, 5] and biological [3] features, which allows for defining a specific subgroup of patients with chronic low back pain (cLBP) [6, 7]. Elementary MRI and histopathological alterations involving intervertebral disc (IVD) and vertebral endplate subchondral bone (VESB) associated with Modic 1 changes, were first described and classified by Modic *et al.*, in the late 1980s, in 474 patients [4, 5]. Briefly, Modic 1 changes are characterized by VESB decreased signal intensity on T1-weighted images and increased signal intensity on T2-weighted images, whereas Modic 2 changes show VESB increased signal intensity on T1-weighted images and isointense or slightly increased signal intensity on T2-weighted images [5]. Along with VESB changes, changes in IVD related to degenerative disc disease (DDD) also occur, and include intervertebral space narrowing (IVSN) and disc herniation [5, 8].

In Modic et al. study, Modic 1 VESB changes were consistently associated with DDD features. Correlation with the grade of DDD was not assessed [5]. Yu *et al.* recently reported that Modic changes were correlated with Pfirrmann grades of lumbar DDD. These grades were significantly higher with Modic 1 than Modic 0 or 2 changes. However, grades I, II and III DDD-associated Modic signals were not described [9]. Only few studies have addressed the spatio-temporal pattern between VESB changes and DDD. In a prospective observational study of 344 people from the Danish general population, who underwent MRI at baseline and at 4-year follow-up, levels with DDD, bulges or herniations predicted new MRI VESB signal changes. Severity of DDD was also associated with the extent of Modic changes [10]. Conversely, in another one-year follow-up prospective study of 54 patients, lumbar levels with Modic 1 changes predicted increased incidence of VESB deformation, IVSN and change in disc signal intensity [11]. Findings from another prospective MRI study of 24 cLBP patients were consistent [12].

Modic changes can also occur with only mild DDD features. In a retrospective study of 150 patients, the prevalence of Modic changes in patients with no IVSN and no hyperostosis was 16.9%, and 29.7% in patients with hyperostosis only [13]. In another study, the prevalence of all Modic changes at the level of a normal IVD was 9.9% [14], while the prevalence Modic 1 changes without IVSN was 29% [12]. Altogether, published data suggest that Modic 1 changes are most frequently associated with advanced DDD, but that they can also occur at the level of a normal IVD or with only mild DDD features. Whether this finding reflects either two different stages of the same dynamic process or two different subsets of Modic 1 changes remain unclear.

We hypothesized that Modic 1 changes associated with less advanced DDD may represent a subset of VESB changes, different from those accompanying advanced DDD. Therefore, we designed a cross-sectional study in order to compare Modic 1 changes associated with advanced DDD to those associated with less advanced DDD.

## Methods

### Ethical considerations

French observational studies of data obtained without any additional therapy or monitoring procedure do not need formal approval from an Institutional Review Board or an Independent Ethics Committee, and formal written consent from the patients is not required for this kind of project. This statement was confirmed by our Institutional Review Board (*Comité de Protection des Personnes – Île-de-France 1*, July 2015) after review of the final version of our manuscript.

### MRI selection

Between January 1, 2006 and January 31, 2008, 1535 consecutive lumbar MR images were recorded in the computerized radiology database of a tertiary care hospital in France specialized in joint, bone, and spine radiology. The strength of the magnet was 1.5 T and the manufacturer of the MRI scanner used was General Electric. MR images were selected for final analysis by using a clinical Picture Archiving and Communication System (PACS) unit. Images were excluded if their computerized record did not contain the key word “spine,” then excluded if the record did not contain the key word “lumbar”. Two assessors screened selected MR images and reviewed patient demographics. Images were excluded if patients were < 18 years old; if MRI did not involve the lumbar spine or showed no Modic 1 changes; or if Modic 1 changes involved 2 levels or only one vertebral endplate. VESB changes related to a specific spine condition were ruled out by reviewing clinical charts. MR images showing Modic 1 changes at a single lumbar level, on both adjacent endplates, were included for further analysis. All 101 examinations included sagittal T2-weighted images with fat signal suppression. Most examinations (83/101) included sagittal T1-weighted images.

### Demographics

Patients’ age and sex were extracted from the radiology computerized database. Other demographical and clinical features including body mass index were not recorded.

### Lumbar MRI assessment

MR images were reviewed in consensus by 2 assessors using a standardized form. The 2 assessors were residents specialized in physical medicine and rehabilitation (MJ) and in rheumatology (CN) with 1-year experience with lumbar MRI interpretation/analysis. Modic 1 signal characteristics and the presence or absence of IVD and VESB changes were assessed as follows:

#### Modic 1 changes

**Fig. 1:**
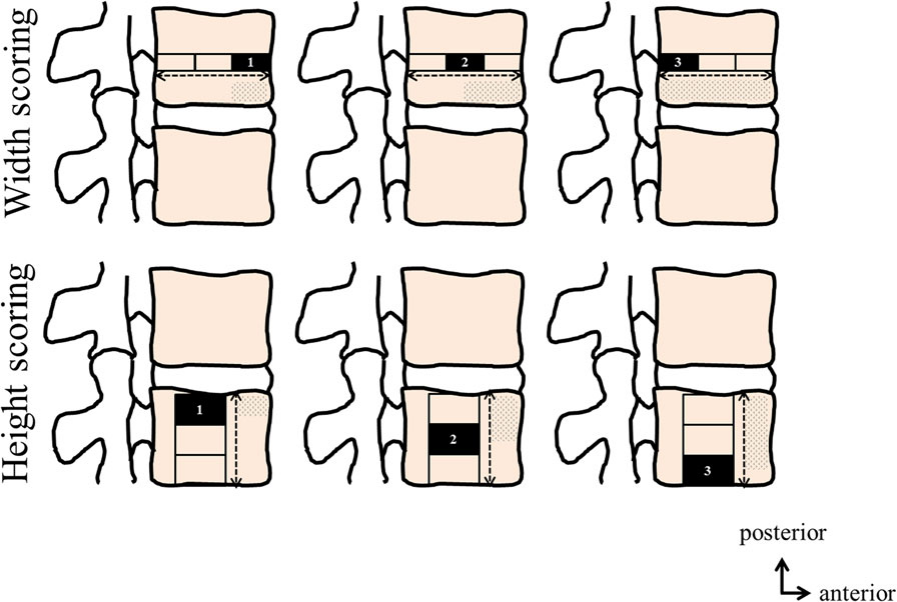
Modic 1 changes width and height semi-quantitative scoring system. Modic 1 signal changes extending to the vertebral body were graded in the cranio-caudal axis for height and in the antero-posterior axis for width, based on the review of all slices, by using a semi-quantitative score ranging from 1 (edema involving < 1/3 of the vertebral body) to 3 (edema involving > 2/3 of the vertebral body)

The lumbar level of Modic 1 signal changes was recorded; Modic 1 signal changes extending to the vertebral body were graded in the cranio-caudal axis for height and in the antero-posterior axis for width, based on the review of all slices, by using a semi-quantitative score ranging from 1 (edema involving < 1/3 of the vertebral body) to 3 (edema involving > 2/3 of the vertebral body), and location of Modic 1 signal changes at the anterior, posterior and/or lateral part of the adjacent vertebral endplate and/or in a periosteophytic region was recorded (Fig. 1).

#### IVD and VESB changes

Global IVD and VESB changes adjacent to Modic 1 changes were reviewed. IVSN was defined as the maximal height of the IVD visually estimated on the mid-sagittal T2-weighted image and described as a percentage of the nearest normal above disc height [15]. Disc height decrease was noted as ≤50% or >50%, which allowed for defining 2 subgroups of patients: >50%-IVSN and ≤50%-IVSN. Other changes noted were: asymmetrical DDD, spondylolisthesis, anterior and posterior IVD herniation, anterior and lateral osteophytes.

### MRI inclusion

**Fig. 2:**
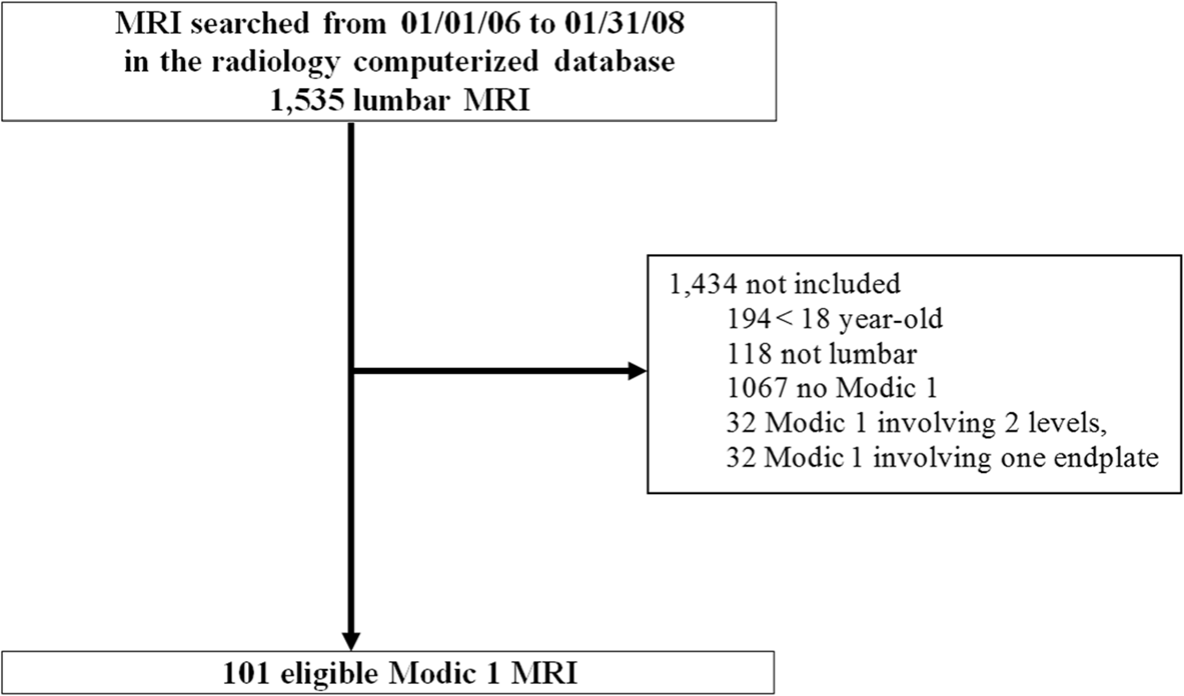
Flow chart of selection of lumbar MRI

Between 2006 and 2008, 1535 lumbar MRI were performed. Overall, 101 MR images showing Modic 1 signal changes at a single lumbar level on both endplates were included in the final analysis (Fig. 2).

### Statistical analysis

Data analysis involved use of Systat v9 (Systat, Chicago, IL). Qualitative data were described with number (percentage) and quantitative data with mean (standard deviation [SD]). For bivariate analysis, comparisons involved nonparametric Kruskal-Wallis test for quantitative variables and nonparametric Fisher’s exact test for qualitative variables. Bonferroni adjustment was used for multiple comparisons (20 comparisons), with *p* < 0.0025 considered statistically significant. Multivariate analysis was conducted to determine factors independently associated with <50%-IVSN Modic 1 changes by backward stepwise regression, with p = 0.10 to enter and p = 0.10 to stay in the model. *P* < 0.05 was considered significant in the final model.

## Results

### Demographics and MRI findings

Patients’ mean (SD) age was 56.6 (13.4) years, and 41 (40.6%) were men (Table 1). Modic 1 signal changes were observed most often at L4/L5 and L5/S1. These levels represented more than two thirds of all Modic 1 cases, 37 (36.6%) cases each. By use of a semi-quantitative scoring system, mean (SD) Modic 1 signal change height was 1.4 (0.6) and width was 2.6 (0.7). In > 50% of the cases, the 5 most frequent structural alterations found at the level of Modic 1 changes were: >50%-IVSN, anterior and posterior IVD herniation, and anterior and posterior osteophytes, in 70/101 (69.3%), 51/101 (50.5%), 56/101 (55.4%), 68/101 (67.3%), and 59/101 (58.4%) cases, respectively (Table 1).

**Table 1:** Demographics and lumbar MRI features of 101 Modic 1 patients

Demographics	
Age, years (SD)	56.6 (13.4)
Male sex	41 (40.6)
Modic 1 changes	
Modic 1 location	
L1/L2 level	1 (1.0)
L2/L3 level	15 (14.9)
L3/L4 level	11 (10.9)
L4/L5 level	37 (36.6)
L5/S1 level	37 (36.6)
Anterior Modic 1	33 (32.7)
Posterior Modic 1	23 (22.8)
Lateral Modic 1	68 (67.3)
Periosteophytic Modic 1	8 (7.9)
Modic 1 extension	
Height, mean (SD)	1.5 (0.6)
Width, mean (SD)	2.6 (0.7)
IVD and VESB changes	
IVSN > 50%	70 (69.3)
Asymmetrical DDD	44 (43.6)
Spondylolisthesis	25 (24.8)
Anterior IVD herniation	51 (50.5)
Posterior IVD herniation	56 (55.4)
Anterior osteophytes	68 (67.3)
Lateral osteophytes	59 (58.4)

Data are no. (%) of patients unless indicated

*DDD* degenerative disc disease, *IVD* intervertebral disc, *IVSN* intervertebral space narrowing, *VESB* vertebral endplate subchondral bone

### ≤50%- and >50%-IVSN Modic 1 changes by demographics and Modic 1 characteristics

Patients with ≤50%-IVSN Modic 1 changes were significantly younger than those with >50%-IVSN changes, and the proportion of men was higher (Table 2). Endplate marrow edema was less extensive with ≤50%- than >50%-IVSN changes (Table 3).

**Table 2:** Demographics by intervertebral space narrowing at the level of Modic 1

	IVSN		
	≤50% (*n* = 31)	>50% (*n* = 70)	*p* value^†^
Age, years (SD)	51.5 (14.1)	58.8 (12.6)	0.019
Male sex, n (%)	17 (54.8)	24 (34.3)	0.078

^†^Nonparametric Kruskal-Wallis test or Fisher’s exact test

**Table 3:** Modic 1 changes characteristics by intervertebral space narrowing at the level of Modic 1

	IVSN		
	≤50% (*n* = 31)	>50% (*n* = 70)	*p* value^†^
Modic 1 location			
L1/L2 level	0 (0.0)	1 (1.4)	1.000
L2/L3 level	1 (3.2)	14 (20)	0.034
L3/L4 level	4 (12.9)	7 (10)	0.733
L4/L5 level	7 (22.6)	30 (42.9)	0.073
L5/S1 level	19 (61.3)	18 (25.7)	0.001*
Anterior Modic 1	13 (41.9)	20 (28.6)	0.169
Posterior Modic 1	6 (19.4)	17 (24.3)	0.797
Lateral Modic 1	24 (77.4)	44 (62.9)	0.107
Periosteophytic Modic 1	4 (12.9)	4 (5.7)	0.245
Modic 1 extension			
Height, mean (SD)	1.4 (0.6)	1.6 (0.6)	0.078
Width, mean (SD)	2.2 (0.8)	2.7 (0.6)	<0.001*

Data are no. (%) of patients unless indicated

^†^Nonparametric Kruskal-Wallis test or Fisher’s exact test; *p < 0.0025

### ≤50%- and >50%-IVSN Modic 1 changes by IVD and VESB changes

**Fig. 3:**
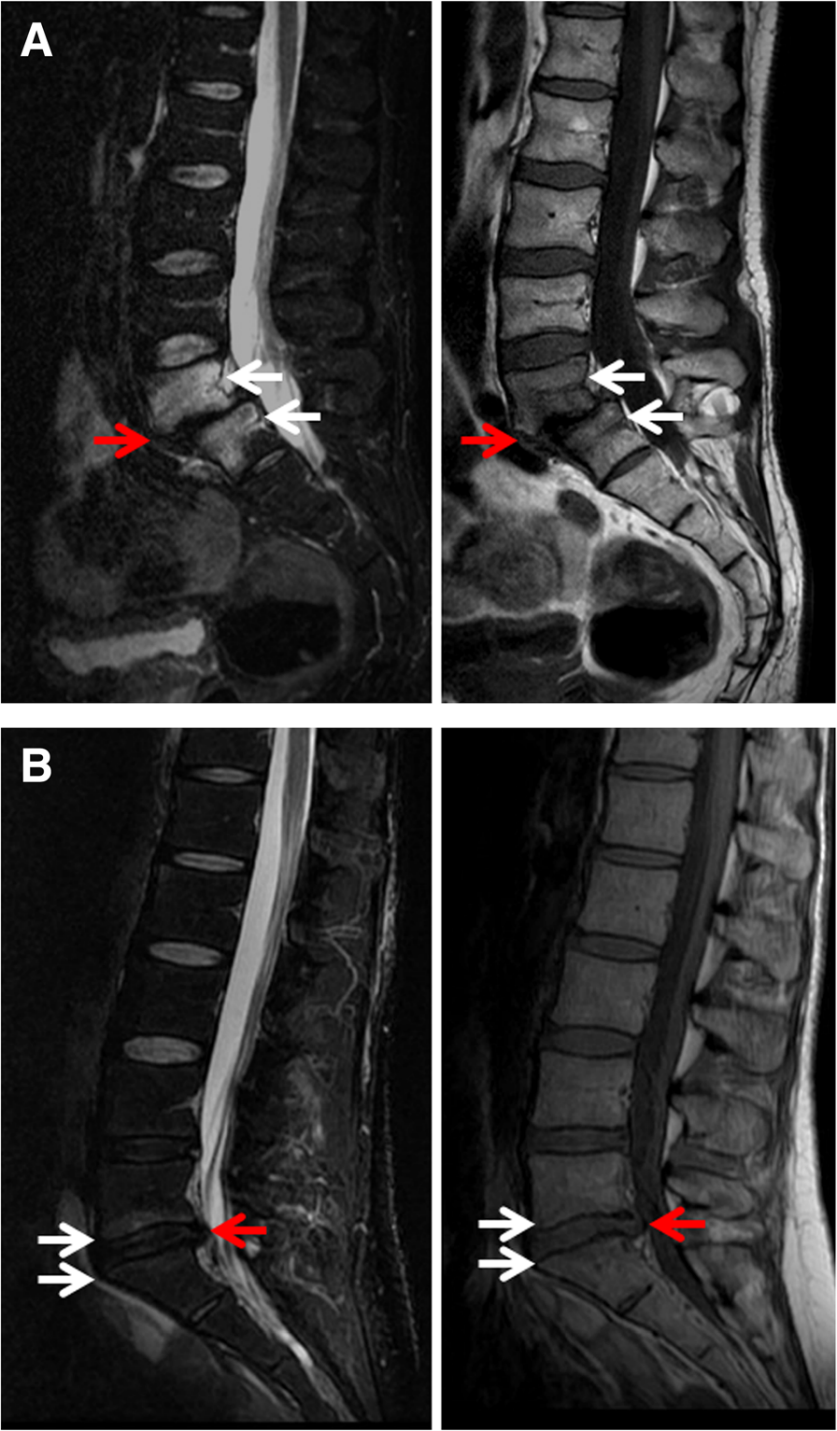
Examples of intervertebral space narrowing >50% intervertebral space narrowing (>50-IVSN) Modic 1 signal changes and ≤50%-IVSN Modic 1 changes on lumbar MRI. **a** >50%-IVSN Modic 1 changes in L5/S1 in a 52-year-old man. Decrease in intervertebral disc height was >50%, bone edema was present and extended to L5 and S1 vertebral bodies (white arrows) with hyperintense signal in T2-weighted sequence (left panel) and hypointense signal in T1-weighted sequence (right panel), and L5/S1 antelisthesis was observed (red arrow); **b** ≤50%-IVSN Modic 1 signal changes in L5/S1 in a 35-year-old woman. Decrease in intervertebral disc height was <50%, edema extended to L5 and S1 vertebral endplates (white arrows) with hyperintense signal in T2-weighted sequence (left panel) and hypointense signal in T1-weighted sequence (right panel), and posterior disc protrusion was observed (red arrow). L4/L5 lumbar DDD was also present

Anterior IVD herniation at the level of Modic 1 changes was less frequent in patients with ≤50%- than >50%-IVSN changes and anterior and posterior osteophytes were less frequent (Table 4) (Fig. 3). On multivariate analysis, variables independently associated with >50%-IVSN Modic 1 changes were location at level L4/L5 and presence of anterior IVD herniation and lateral osteophytes (Table 5).

**Table 4:** Intervertebral disc and vertebral endplate subchondral bone changes by intervertebral space narrowing at the level of Modic 1

Structural changes	IVSN		
	≤50% (*n* = 31)	>50% (*n* = 70)	*p* value^†^
Asymmetric discopathy	10 (32.3)	34 (48.6)	0.191
Spondylolisthesis	4 (12.9)	21 (30.0)	0.129
Anterior disc herniation	11 (35.5)	40 (57.1)	0.054
Posterior disc herniation	16 (51.6)	40 (57.1)	0.667
Anterior osteophytes	13 (41.9)	55 (78.6)	<0.001*
Posterior osteophytes	11 (35.5)	48 (68.6)	0.002*

Data are no. (%) of patients

^†^Nonparametric Kruskal-Wallis test or Fisher’s exact test; *p value < 0.0025

**Table 5:** Multivariate analysis of factors associated with > 50%-IVSN Modic 1 signal changes detected on lumbar MRI

	Adjusted OR	95% CI	*p* value
Modic 1 signal at L4/L5	0.191	0.060-0.610	0.005*
Anterior disc herniation	0.205	0.060-0.607	0.004*
Posterior osteophytes	0.268	0.095-0.753	0.012*

*OR* odds ratio, *CI* confidence interval

Adjusted for: age, sex, Modic 1 height and width

*p value < 0.05

## Discussion

In our cross-sectional study of 101 Modic 1 changes involving the lumbar level, we compared IVD and VESB changes associated with advanced DDD to those associated less advanced DDD. We found that ≤50%-IVSN Modic 1 were rather found in young men at L5/S1 level and are associated with less frequent osteophytes, while >50%-IVSN Modic 1 were rather found in older women at L4/L5 level.

IVSN has been considered a hallmark of DDD [5, 16]. Its frequency, when Modic changes are present, is up to 90.1% [14]. Some authors reported Modic changes without or with minimum IVSN [12–14]. The prevalence of this phenotype ranges from 9.9% [14] to 29.7% [13]. Variability between studies is due in part to heterogeneous definition and assessment methods of IVSN and to the absence of analysis by Modic change type. No studies have described the demographic and imaging phenotype in these patients. In our study, Modic 1 patients with relatively conserved disc height differed, namely ≤50%-IVSN, significantly differed from those with >50%-IVSN, and overall from Modic 1 population described in the literature [17]. ≤50%-IVSN Modic 1 patients were younger and the men to women ratio was higher than those with >50%-IVSN. In addition, ≤50%-IVSN Modic 1 changes were less often associated with osteophytes and more often located at L5/S1 level, as compared with >50%-IVSN Modic 1 changes, which were more frequent at L4/L5 level. On multivariate analysis, predictors of >50%-IVSN Modic 1 changes were location at L4/L5 level, and variables reflecting more advanced DDD, such as anterior disc herniation and lateral osteophytes. Osteophyte formation has been described by some authors as a compensation mechanism to distribute the increasing axial and shear forces (due to instability) on a larger articulating surface [18]. Therefore, one can assume that differences in osteophyte formation between >50%- and ≤50%-IVSN Modic 1 might reflect differential biomechanical factors. Current hypotheses of Modic 1 changes include biochemical and biomechanical stress [19], along with local infection [20–22] and genetic predisposition [23, 24]. ≤50%-IVSN and >50%-IVSN Modic 1 groups, may reflect successive stages of the same dynamic process. However, based on the specific demographic findings of our study, one can also hypothesize, that these two groups might represent distinct subsets of Modic 1 patients, that might involve different physiopathological processes. The cross-sectional design of our study did not allow to further address this hypothesis. Proper phenotyping of Modic 1-associated cLBP patients may help guide clinical decisions and develop more targeted treatments [7].

Our study had several limitations. Our sample size was small. Because some events were rarely observed, our statistical analysis lacked power. However, because we focused on only Modic 1 changes, our sample of ≤50%-IVSN Modic 1 changes was larger than in previously published studies and allowed us to compare this subgroup of Modic 1 patients to those with more advanced DDD stages. As well, cases were selected solely from a systematic screening of a computerized database, regardless of clinical presentation. Therefore, we could not address clinical correlates of the MRI phenotype. In addition, MRI cases were from a tertiary care radiology facility and not representative of the whole French primary care population, because patients were more likely referred for a specific condition or symptom. A more comprehensive classification of DDD would probably have been obtained by using the Pfirrmann grading system of lumbar DDD [16]. However, we focused on only some DDD features that we thought were relevant to address our hypothesis. Our method to assess IVSN had some limitations. Indeed, we assessed decreased disc height only on the mid-sagittal view. Methods to quantify IVD height have not been standardized, and they especially do not take into account more localized IVSN. Some authors have recently developed the disc height index to objectively quantify disc height. However, this index needs further validation [25]. Images were analyzed in consensus by the 2 assessors. They should have ideally been read independently. Finally, the cross-sectional design of our study did not allow for addressing the predictive value of observed changes on clinical and structural outcomes, namely, accelerating the DDD process and cLBP symptoms.

## Conclusions

≤50%-IVSN Modic 1 patients differ from >50%-IVSN Modic 1 patients on some demographic and IVD and VESB features. Overall, these patients were younger, more often men, had less advanced DDD features, and changes were more often located at L5/S1 level. Whether these findings reflect either two different stages of the same dynamic process or two phenotypically distinct subsets of Modic 1 changes has to be further assessed.

## Abbreviations

cLBP: Chronic low back pain
DDD: Degenerative disc disease
IVD: Intervertebral disc
IVSN: Intervertebral space narrowing
MRI: Magnetic resonance imaging
SD: Standard deviation
VESB: Vertebral endplate subchondral bone
